# M6a demethylase FTO regulates the oxidative stress, mitochondrial biogenesis of cardiomyocytes and PGC-1a stability in myocardial ischemia-reperfusion injury

**DOI:** 10.1080/13510002.2025.2454892

**Published:** 2025-01-27

**Authors:** Qiong Jiang, Xuehai Chen, Kezeng Gong, Zhe Xu, Lianglong Chen, Feilong Zhang

**Affiliations:** a Department of Cardiology, Fujian Medical University Union Hospital, Fuzhou, Fujian, People’s Republic of China; b Fujian Institute of Coronary Heart Disease, Fuzhou, Fujian, People’s Republic of China; c Fujian Heart Medical Center, Fuzhou, Fujian, People’s Republic of China

**Keywords:** FTO, oxidative stress, mitochondrial biogenesis, PGC-1a, myocardial ischemia-reperfusion injury

## Abstract

**Objective:**

Myocardial ischemia-reperfusion injury (MIRI) is a highly complex disease with high morbidity and mortality. Studying the molecular mechanism of MIRI and discovering new targets are crucial for the future treatment of MIRI.

**Methods:**

We constructed the MIRI rat model and hypoxia/reoxygenation (H/R) injury cardiomyocytes model. RT–PCR and Western blot were used to investigate the expression of the fat mass and obesity-associated (FTO) gene. Electrocardiogram, echocardiography, triphenyltetrazolium chloride (TTC) staining and hematoxylin-eosin (HE) staining were used to assess the model and the effect of FTO overexpression. The generation of reactive oxygen species (ROS) and the levels of superoxide dismutase (SOD2), mitochondrial transcription factor (TFAM) and cytochrome c oxidase I (COXI) were detected to assess the oxidative stress and mitochondrial biogenesis. RNA immunoprecipitation (RIP) and RNA pulldown assays were used to identify the interaction of FTO and PGC-1a. The m6A dot blot, methylated RNA immunoprecipitation PCR (MeRIP-PCR) and RNA stability analysis were used to analyze the regulation of methylation of PGC-1a by FTO.

**Results:**

FTO was downregulated in MIRI rats and H/R induced cardiomyocytes. Overexpression of FTO inhibited ROS level and increased the expression of SOD2, TFAM and COXI in vitro and in vivo. In addition, PGC-1a was identified as a downstream target of FTO. FTO enhanced the stability of PGC-1a mRNA through removing the m6A modification.

**Conclusion:**

Our study revealed the role of FTO regulates the oxidative stress and mitochondrial biogenesis via PGC-1a in MIRI, which may provide a new approach to mitigating MIRI.

## 1. Introduction

Myocardial ischemia stands as the prevalent type of cardiovascular disease, accounting for a significant portion of morbidity and mortality [1]. Myocardial ischemia-reperfusion injury (MIRI) refers to the secondary damage in the heart ischemia after reperfusion [2]. During the ischemic phase, stenosis or obstruction of the coronary arteries impedes the delivery of adequate oxygen and nutrients to myocardial tissues, resulting in an energy deficit for cardiomyocytes and consequently impairing their function [3]. Reperfusion injury occurs as a biological response to the restoration of blood supply, leading to further damage to the cardiomyocytes [4]. MIRI is associated with ventricular remodeling, cardiac dysfunction, and potentially heart failure, making it a significant determinant of patient prognosis [5]. Despite clinical efforts, no specific treatment exists for MIRI [6]. The current understanding of its pathogenesis involves oxidative stress, calcium overload, mitochondrial dysfunction, inflammation and metabolism disorder [6,7]. Nevertheless, due to its complexity, the precise mechanisms underlying MIRI remain incompletely understood. Thus, elucidating the molecular mechanisms of MIRI is imperative for developing targeted therapeutic approaches.N6-methyladenosine (m6A) is the predominant form of RNA methylation, catalyzed by the methyltransferase complex (writers) to add methyl groups, which are recognized by downstream methyl-recognition proteins (readers) to carry out specific functions, and can be removed by demethylases (erasers) [8]. This dynamic and reversible modification regulates gene expression by affecting RNA stability, splicing, translation efficiency, and degradation [9,10]. Recent investigations demonstrated that m6A modification played a critical role in modulating key genes involved in oxidative stress, inflammation, and mitochondrial function in MIRI [11–13]. Notably, Fat-mass and obesity associated gene (FTO) is an RNA demethylase that plays a crucial role in the regulation of m6A RNA methylation. It reverses the methylation process by removing the m6A modification from RNA, thus influencing RNA stability, translation, and downstream biological functions [14]. Research on FTO’s roles in cardiovascular diseases, particularly in myocardial fibrosis, heart failure, atherosclerosis, and myocardial ischemia, has gained momentum, revealing its various biological effects in the pathophysiology conditions [15]. Recent report had shown that FTO expression was downregulated in MIRI both in vivo and in vitro, and that FTO exerted its protective effects by demethylating yes-associated protein 1 (YAP1) mRNA, enhancing its stability, and thereby promoting cardiomyocyte survival under ischemic conditions [16]. Enhancing FTO expression in heart failure mouse reduced the ischemia-triggered rise in m6A levels and the decline in cardiac contractile function, which selectively demethylates cardiac contractile mRNAs, preventing the degradation and enhancing protein expression during ischemia [17]. FTO decreased the stability of Cbl Proto-Oncogene (CBL) mRNA by inhibiting m6A modification and the CBL-mediated ubiquitination and subsequent degradation of β-catenin were implicated in the suppression of pyroptosis induced by FTO during myocardial ischemia/reperfusion (I/R) injury [18]. However, the specific mechanisms underlying the regulation of oxidative stress and mitochondrial function by FTO in MIRI remain unclear.

Studies have shown that peroxisome proliferator-activated receptor-gamma coactivator-1alpha (PCG-1α) serves as a pivotal transcriptional co-regulator implicated in mitochondrial biogenesis, metabolic processes, and antioxidant defenses, potentially mitigating mitochondrial reactive oxygen species (ROS) levels [19,20]. A reduction in PGC-1α expression is associated with the exacerbation of various metabolic disorders due to elevated ROS levels. Nuclear respiratory factor 2 (Nrf2) and PGC-1α synergistically enhance the transcriptional activity of mitochondrial transcription factor A (TFAM), which directly facilitates the transcription and replication of the mitochondrial genome [21]. PGC-1α was involved in the process of hydrogen reducing apoptosis, oxidative stress, and inflammation in MIRI and also regulated mitochondrial fission/fusion and protects mitochondrial function [22]. The PGC-1α agonist ZLN005 mitigates the cardiomyopathy phenotype by strengthening redox balance, reducing doxorubicin-induced oxidative stress, preventing harmful tissue remodeling, and inhibiting necroptosis [23]. Further investigation is warranted to determine whether FTO influences mitochondrial function regulation in MIRI cardiomyocytes by modulating the m6A modification of PGC-1α.

In this study, we aimed to investigate the role of FTO in mediating MIRI in rat models, as well as in cardiomyocytes subjected to hypoxia/reoxygenation (H/R) injury. We focused on understanding how FTO influences oxidative stress during MIRI, and examined the underlying molecular mechanisms by which FTO modulates the m6A modification of PGC-1α, which could provide valuable insights into its role in MIRI condition.

## 2. Materials and methods

### 2.1. Rat MIRI procedure

Adult male Sprague Dawley (SD) rats (250–300 g) were provided by Anburui Biotechnology Co., Ltd (Fuzhou, Fujian, China). Rats were fed a standard diet for 1 week of adaptive feeding. The rats were randomly divided into three groups: sham group (*N* = 6), MIRI (*N* = 6) and MIRI + FTO overexpression (FTO-OE) (*N* = 6). In brief, following anesthesia achieved via an intraperitoneal injection of 60 mg/kg pentobarbital sodium, the left thorax was surgically opened to access the heart. Subsequently, the left anterior descending coronary artery (LAD) was occluded using a sterile 7/0 suture to induce myocardial ischemia. This ischemia was maintained for 30 min, followed by 2 h of reperfusion. The sham group underwent an identical surgical protocol, excluding the ligation of the LAD coronary artery. The rats in FTO-OE group were injected with AAV9 vector carrying FTO at a dose of 1 × 10^11^ viral genome particles/rat around the infarction region. Rats were sacrificed by overdose anesthesia after reperfusion for 2 hours. All animal experiments were approved by the Ethics Committee of Anburui Biotechnology Co., Ltd (IACUC FJABR 2023026006).

### 2.2. Electrocardiogram (ECG) assessment

The ECG changes of sham group, MIRI model group or MIRI + FTO-OE group rats were evaluated. Anesthetize all rats during the surgical ligations and assess standard limb lead II tracing to identify ECG changes and confirm myocardial ischemia as a previous study [20].

### 2.3. Echocardiography

The mice cardiac structure and function were evaluated by echocardiography with the Vevo2100 system (Fujifilm Visual Sonics, Toronto, Canada). First, mice were anesthetized with 3% isoflurane and maintained under anesthesia with 1.5% isoflurane. Then, the cardiac function parameters, including ejection fraction (EF), fractional shortening (FS) were measured.

### 2.4. Triphenyltetrazolium chloride (TTC) staining

TTC staining works by detecting the viability of tissue based on metabolic activity. In healthy, viable tissue, the enzyme dehydrogenase reduces TTC to red formazan, leading to a red or pink color. In infarcted (dead) tissue, due to lack of enzyme activity from cell death, the TTC remains unreduced and appears pale or white, allowing clear differentiation between viable and infarcted tissue. The myocardial tissues of rats were taken and rapidly frozen at −20°C for about 20 min for easy slicing. Sequential sections were obtained at regular 1 mm intervals. The slices were placed in TTC (#A610558-0005, Sangon Biotech, Shanghai, China) with a concentration of 2% at 37°C in night for 20 min. Following this, the slices were fixed in 4% paraformaldehyde (#P0099-100 ml, Beyotime, Shanghai, China) for 24 h and photographed for documentation.

### 2.5. Hematoxylin and eosin (H&E) staining

The heart tissues of rats underwent fixation in 4% paraformaldehyde for a duration of 24 hours. Following fixation, the samples were processed for paraffin embedding and subsequently sliced into 4 μm thick sections. For histopathological evaluation, these sections were stained utilizing the hematoxylin–eosin (#G1120, Solarbio, Beijing, China) method in accordance with the standard protocol. Under a light microscope (IX73, Olympus Corporation, Tokyo, Japan), five randomly chosen fields were examined to assess any histological alterations.

### 2.6. Cell culture and hypoxia/reoxygenation (H/R) injury treatment

The H9C2 rat cardiomyocytes, procured from ATCC (Manassas, VA, USA), were grown in a DMEM/high glucose medium (#11965092, Thermo Fisher, Waltham, MA, USA) supplemented with 10% FBS (#A5670701, Thermo Fisher, Waltham, MA, USA) and 1% penicillin–streptomycin (#C0222, Beyotime, Shanghai, China). To model a hypoxic/reoxygenation (H/R) scenario, the cardiomyocytes were subjected to hypoxic conditions (1% O_2_, 5% CO_2_, 94% N_2_) for 12 h. Following this hypoxic period, the cells were then incubated in a normoxic environment (95% air, 5% CO_2_) at 37°C for 24 h to mimic reoxygenation. The oxygen levels within the incubator were confirmed using a specialized oxygen sensor (BioSpherix, Redfield, NY, USA).

### 2.7. Flow cytometry detection of ROS

DCFH-DA is sensitive to a variety of ROS, including superoxide (O₂^−^), hydrogen peroxide (H₂O₂), hydroperoxides, and other reactive species, making it a versatile indicator of oxidative stress. DCFH-DA (#HY-D0940, MedChemExpress, Shanghai, China) was diluted with serum-free culture medium at a ratio of 1:1000 to a final concentration of 10 μM. This diluted DCFH-DA solution was then introduced to the heart tissue homogenate or cardiomyocytes. The mixture was incubated in a 37°C incubator in a dark environment for 30  minutes, with periodic agitation every 5 min to promote thorough probe-cell interaction. Afterward, the samples were rinsed three times with serum-free culture medium. Finally, the treated samples were analyzed using flow cytometry, with excitation and emission wavelengths set at 488 and 525 nm, respectively.

### 2.8. Cell transfection

FTO overexpression plasmid (FTO-OE) and negative control were subcloned into the vector pcDNA3.1 (Invitrogen, Carlsbad, CA, USA), generating the vector FTO-OE. The transfection plasmid used in this study was constructed by OBiO Technology Co., Ltd. (Shanghai, China). Plasmids were transfected into the cardiomyocytes using Lipofectamine 2000 (#11668030, Invitrogen, Carlsbad, CA, USA) following the manufacturer’s protocol. The lentivirus packaging for FTO overexpression using AAV9 vector was performed by Gene Pharma (Shanghai, China).

### 2.9. Cell counting kit-8 (CCK-8) assay

The H9c2 cells were seeded onto a 96-well plates (1 × 10^4^ cells/well). At 24 h after post-seeding, 10 μl CCK-8 solution (#C0038, Beyotime, Shanghai, China) was separately added to each well. They were then incubated in a 5% CO_2_ incubator for 1 h at 37°C. The absorbance at 450 nm was recorded using a plate reader (UV-1780, Shimadzu, Japan). Each test was independently repeated at least three times.

### 2.10. Quantitative real-time PCR (RT–PCR)

Total RNA from cardiomyocytes or heart tissues was isolated employing TRIzol reagent (#15596018CN, Invitrogen, Carlsbad, CA, USA). Subsequently, this RNA was converted to cDNA using the PrimeScript™ RT Reagent Kit from Takara (#RR037A, Dalian, China). Quantitative reverse transcription PCR (QRT-PCR) was performed with SYBR Green PCR Mix Kit (#RR067A, Takara, Dalian, China), following the manufacturer’s protocol. GAPDH served as the reference gene for normalization. The relative expression levels of target genes were determined using the 2^−ΔΔCt^ method. The primers sequences (5′−3′) were as follow: GAPDH: F: TGGAAAGCTGTGGCGTGATG, R: TACTTGGCAGGTTTCTCCAGG; FTO: F: CTGTGTTTTGGCTGGCTCAC, R: TCGCCATTGTCCGAGTCATT; SOD2: F: TTGGCCGTACTATGGTGGTC; R: TGGGCAATCCCAATCACACC; PGC-1a: F: CGCTGCTCTTGAGAATGGATAT; R: GTCATACTTGCTCTTGGTGGAA; TFAM: F: CGGCAGAAACGCC TAAAGAAG; R: GCTTCAATTTCCCCTGAGCTG; COXI: F: 5′-GCCGGATTGGTGGGGGTA G-3′; R: 5′-AGGGGCAGGTCTTGGTGT TG-3′.

### 2.11. Western blot

The heart tissues and cardiomyocytes were prepared using RIPA buffer (#R0278, Sigma-Aldrich, Shanghai, China). Protein concentrations were determined using a BCA protein assay kit (#23227, Thermo Fisher, Waltham, MA, USA). Equivalent protein amounts were resolved by sodium dodecyl sulfate-polyacrylamide gel electrophoresis and transferred to PVDF membranes (#IPVH00010, Millipore, Billerica, MA, USA). Prior to antibody incubation, membranes were blocked with 5% skim milk. The membranes were then incubated overnight at 4°C with primary antibodies specific to SOD2 (1:5000, ab13533, Abcam, Shanghai, China), TFAM (1:1000, ab252432, Abcam, Shanghai, China), COXI (1:1000, 4841, Cell Signaling Technology, Danvers, MA, USA), FTO (1:2000, #27226-1-AP, Proteintech, Wuhan, China), PGC-1α (1:10000, 66369-1-lg, Proteintech, Wuhan, China), β-actin (1:2000, ab8227, Abcam, Shanghai, China)and GAPDH (#GB15002, 1:2000, Servicebio, Wuhan, China). The following day, membranes were probed with horseradish peroxidase-conjugated secondary antibodies (#SA00001-2 & SA00001-1, 1:3000, Proteintech, Wuhan, China) for 1 hour at room temperature. Finally, protein bands were visualized using an enhanced chemiluminescence system (MA0186-1, Meilunbio, Dalian, China).

### 2.12. RNA pulldown

To investigate the potential interaction between FTO and PGC-1a, a RNA pull-down experiment was conducted utilizing the Pierce Magnetic RNA-Protein Pull-Down Kit (#20164, Thermo Scientific, Waltham, MA, USA). Briefly, biotinylated RNA was combined with protein lysates extracted from H9c2 cells and streptavidin-conjugated magnetic beads. After thorough washing, the beads were analyzed by immunoblotting to identify the captured proteins.

### 2.13. RNA immunoprecipitation (RIP)-PCR

RIP was carried out with the Magna RIP RNA-Binding Protein Immunoprecipitation Kit (#17-700, Millipore, Billerica, MA, USA). Cardiomyocytes were lysed using RIP lysis buffer. Anti-FTO antibodies were incubated with protein A/G agarose beads coupled to specific antibodies or control IgG at 4°C for 2 h. After washing the magnetic beads and treating them with proteinase K, RNA was extracted from the RNA-protein complexes and evaluated using RT–PCR.

### 2.14. Methylated RNA immunoprecipitation PCR (MeRIP-PCR)

To assess m6A modification, the MeRIP m6A Transcriptome Profiling Kit (#C11051-1, RiBoBio, Guangzhou, China) was employed. Initially, total RNA was isolated using TRIzol reagent (#15596018CN, Invitrogen, Carlsbad, CA, USA) and fragmented for 3 min at 94°C. Magnetic beads A/G were then incubated with 5 μg of anti-m6A antibody for 30 min under rotation. After washing three times, the fragmented RNA was subjected to immunoprecipitation with m6A or IgG antibody-conjugated beads for 2 h at 4°C. The samples were eluted in an elution buffer, and enrichment of m6A was subsequently analyzed by RT–PCR.

### 2.15. M6a dot blot analysis

Total RNA was normalized to a standard concentration of 600 ng/μL or 300 ng/μL and heated in a metal bath at 95°C for 5 minutes. Following this, the solution was chilled on ice for 10 minutes until completely cooled. RNA was then mixed with 20× saline sodium citrate in a 1:1 ratio. Samples were loaded onto a nitrocellulose filter membrane in a concentration gradient, and a UV adhesive was applied for 15 min. The bonded nitrocellulose filter was immersed in aqua blue solution for 2 min, washed with TBS-T for cleaning, and prepared for subsequent imaging. The subsequent procedures mimicked those of western blot analysis.

### 2.16. RNA stability assay

Cardiomyocytes were plated in 6-well dishes (1 × 10^5^ cells per well). Following 24 h, they were treated with 2 μg/ml of Actinomycin D (ActD, #A1410, Sigma-Aldrich, Shanghai, China) and collected at designated intervals (3 and 6 h). The stability of RNA was assessed by RT–PCR, measuring its remaining level and normalizing it to the 0 h.

### 2.17. Statistical analysis

Statistical analyses were performed using GraphPad Prism (V9.0.1, GraphPad, USA). All data are presented as mean ± standard deviation (SD). Comparisons between two groups were performed using the Student’s t-test, while comparisons among three or more groups were conducted using one-way analysis of variance (ANOVA) followed by Tukey’s multiple comparisons test. *p*-value < 0.05 was deemed significant.

## 3. Results

### 3.1. Construction of myocardial ischemia-reperfusion injury rats

**Figure 1.**
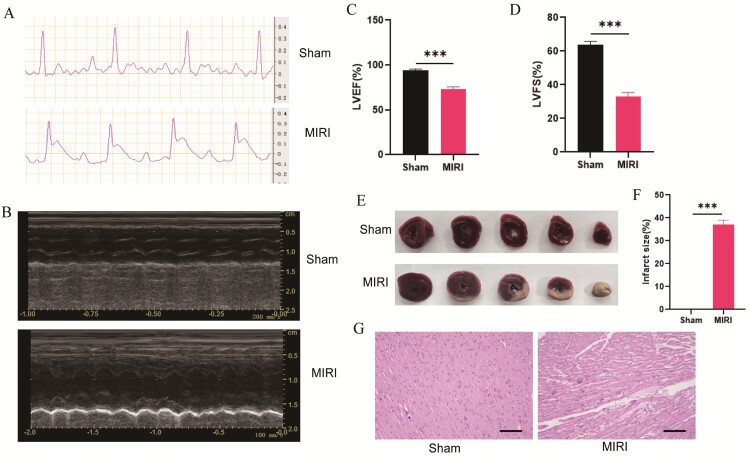
Construction of myocardial ischemia-reperfusion injury rats. (A)The MIRI rats showed a higher ST-segment elevation than the sham group (*N* = 6). (B) Echocardiography was performed to test heart function of rats in sham group, MIRI model group (*N* = 6). (C–D) Ejection fraction, fractional shortening of the left ventricular diameter (*N* = 6). (E) Heart sections of rats from the two groups were stained with TTC staining (*N* = 3). (F) The infarct size differences between the MIRI model group and sham group rats (*N* = 3). (G) H&E staining of the heart sections of the sham and MIRI rats (*N* = 3) (scale bar = 100 µm). ***p < 0.001. All the data were presented as the means ± SD.

The ECG ST segments of rats in experimental MIRI model groups were elevated compared with those of rats in the sham group **(**Figure 1**(**A)). Then, the echocardiography (Figure 1**(**B)) showed that cardiac function worsened in MIRI rats compared to sham group with a significant decrease in the ejection fraction (EF%) and fractional shortening (FS%) of the rats in the MIRI group compared with those in the sham group (Figure 1**(**C,D)**)**. In addition, heart sections of rats from the two groups were stained with TTC. Compared to the sham group, the MIRI rats had myocardial infarctions (Figure 1**(**E)) and the infarct size differences between the MIRI model and sham group were significant (Figure 1**(**F)). Further, HE staining results showed that the cardiomyocytes of MIRI group had sustained some degree of damage and were infiltrated by some neutrophils compared to the sham group (Figure 1**(**G)).

### 3.2. FTO was down-regulated in MIRI rats and H/R-induced cardiomyocytes

**Figure 2.**
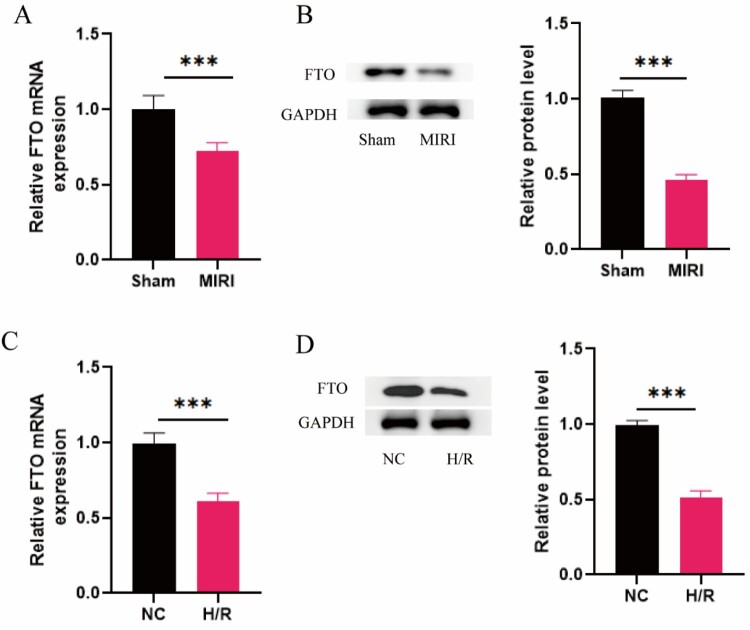
FTO was down-regulated in MIRI rats and H/R-induced cardiomyocytes. (A) FTO mRNA expression in MIRI and Sham rats (*N* = 3). (B) Western blot was conducted to access the protein expression of MIRI and Sham rats (*N* = 3). (C) FTO mRNA expression in H/R-induced and normal H9C2 cells (*N* = 3). (D) Western blot was conducted to access the protein expression of FTO in H/R-induced and normal H9C2 cells (*N* = 3)**.** ***p < 0.001. All the data were presented as the means ± SD.

To investigate the regulatory role of m6A-related protein FTO in MIRI, we detected the expression of FTO in MIRI rats and H/R-induced cardiomyocytes. Results showed that FTO mRNA was downregulated in MIRI tissues (Figure 2**(**A)). As FTO plays a role in regulating m6A modification at the protein level, the protein expression level of FTO in MIRI tissues was also investigated. The results showed that FTO exhibited lower protein expression in MIRI tissues (Figure 2**(**B)). Further, in H/R-induced cardiomyocytes, the mRNA and protein expression levels of FTO showed the same tendency (Figure 2**(**C, D)).

### 3.3. Overexpression of FTO inhibits excessive oxidative stress and restores mitochondrial biogenesis in H/R cardiomyocytes

**Figure 3.**
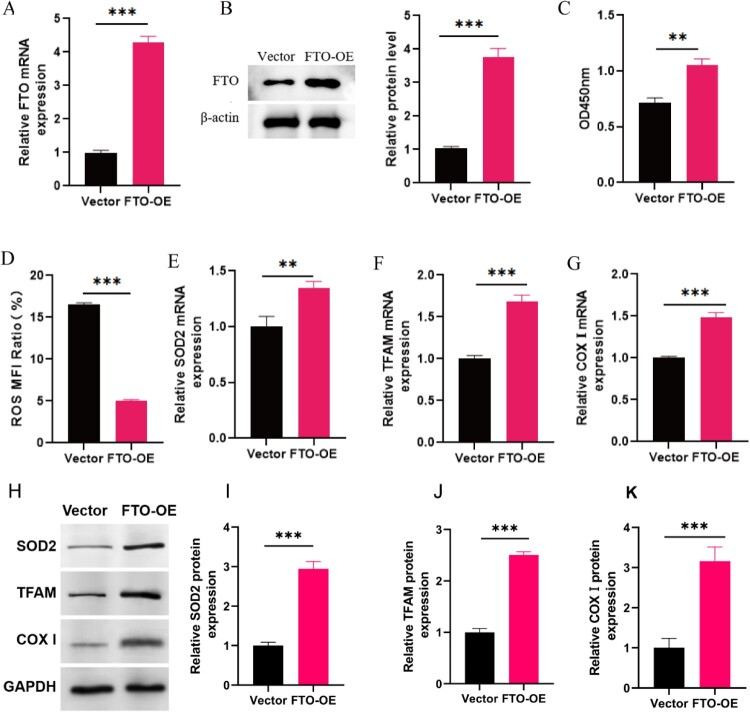
Overexpression of FTO inhibits excessive oxidative stress and restores mitochondrial function in H/R cells. (A–B) RT-PCR and western blot were used to detect the mRNA and protein expression level in H/R (Vector) and H/R + FTO-OE cardiomyocytes (*N* = 3). (C) CCK8 assay showed the cell proliferation of H/R (Vector) and H/R + FTO-OE cardiomyocytes (*N* = 3). (D)The ROS level in H/R (Vector) and H/R + FTO-OE cardiomyocytes (*N* = 3). (E) RT-PCR showed the expression of SOD2 (*N* = 3). (F) The expression of mitochondrial transcription factor TFAM and mtDNA marker gene COXI (*N* = 3). (H) The protein expressions of SOD2, TFAM and COXI determined by Western blot assay (*N* = 3). (I–K) Quantitative analysis of expression level of SOD2, TFAM and COXI (*N* = 3). **p < 0.01, ***p < 0.001. All the data were presented as the means ± SD.

Then, we investigated the functions of FTO in H/R-induced H9C2 cardiomyocytes. The RT–PCR and western blot showed that FTO mRNA and protein levels were both upregulated in H9C2 cells FTO overexpression (FTO-OE) group (Figure 3**(**A,B)). The CCK8 assay indicated that FTO overexpression promoted the cell proliferation (Figure 3**(**C)). In addition, we found that FTO can reduce the ROS level and increase the expression of SOD2 (Figure 3**(**D, E)). Further, mitochondrial transcription factor TFAM and COXI gene encoded by mtDNA were both upregulated in FTO overexpression group (Figure 3**(**F, G)). In addition, the results of Western blot confirmed that FTO overexpression significantly promoted the protein expressions of SOD2, TFAM and COXI (Figure 3**(**H–K)). These results indicated that FTO inhibits excessive oxidative stress and restores mitochondrial function in H/R cardiomyocytes.

### 3.4. Overexpression of FTO inhibits excessive oxidative stress and restores mitochondrial biogenesis in MIRI rats

**Figure 4.**
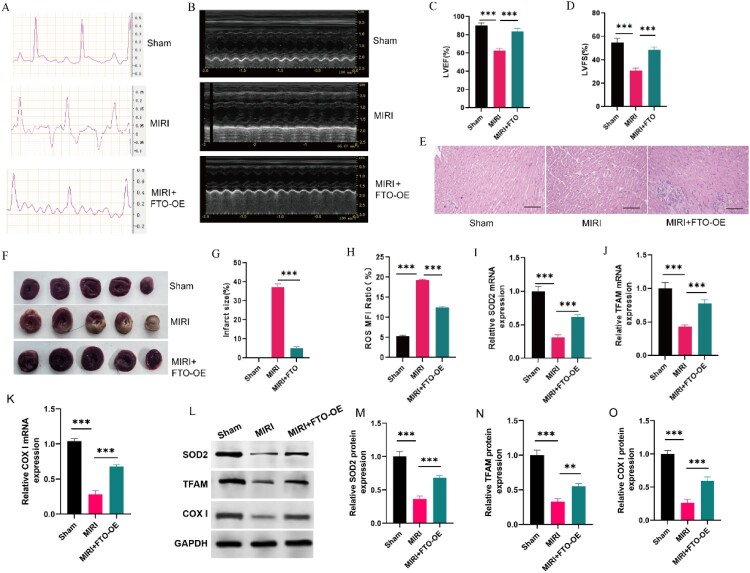
Overexpression of FTO inhibits excessive oxidative stress and restores mitochondrial function in MIRI rats. (A) Electrocardiogram was performed to test heart function of the rats (*N* = 6). (B) Echocardiography was performed to test heart function of sham, MIRI, and MIRI + FTO-OE rats (*N* = 6). (C–D) Ejection fraction, fractional shortening of the left ventricular diameter (*N* = 6). (E) H&E staining of the heart sections of the rats (*N* = 3) (scale bar = 100 µm). (F) Heart sections of rats from the three groups were stained with TTC staining (*N* = 3). (G) The infarct size differences among the three group rats (*N* = 3). (H)The ROS level in H/R (Vector) and H/R + FTO-OE cardiomyocytes (*N* = 3). (I) RT-PCR showed the expression of SOD2 (*N* = 3). (J) The expression of mitochondrial transcription factor TFAM (*N* = 3). (K) RT-PCR detected the expression of COXI mRNA level (*N* = 3). (L) The protein expressions of SOD2, TFAM and COXI determined by Western blot assay (*N* = 3). (M-O) Quantitative analysis of expression level of SOD2, TFAM and COXI (*N* = 3). ***p < 0.001. All the data were presented as the means ± SD.

Subsequently, we investigated the functions of FTO in MIRI rats. The ECG ST segments of rats in the FTO group were relatively normal compared with those of rats in the MIRI group (Figure 4**(**A)). Then, we assessed the heart function of the mice using echocardiography (Figure 4**(**B)). Our results showed a significant decrease in the LVEF and LVFS of the rats in the MIRI group compared with those in the sham group, but this reduction was completely restored in the FTO-OE group (Figure 1**(**C,D)). In addition, HE staining results showed that the cardiomyocytes of FTO-OE group showed better integrity compared to the MIRI group (Figure 4**(**E)). Otherwise, heart sections of rats stained with TTC showed that the FTO-OE group rats has much smaller myocardial infarctions than the MIRI rats (Figure 4**(**F,G)). What’s more, we found that FTO overexpression can reduce the ROS level and increase the expression of SOD2 in the myocardial tissues (Figure 4**(**H, I)). Further, mitochondrial transcription factor TFAM and COXI gene encoded by mtDNA were upregulated in FTO overexpression group (Figure 4**(**J,K)). Additionally, Western blot assay was conducted and the results were consistent with those of RT–PCR analysis (Figure 4**(**L–O)). These results indicated that FTO overexpression may inhibit oxidative stress and restore mitochondrial function in MIRI rats.

### 3.5. Identification of PGC-1a as a downstream target of FTO

Next, we tried to investigate the downstream of FTO through which FTO exerted its functions in MIRI. We tried to investigate the interaction within FTO and PGC-1a in MIRI.

**Figure 5.**
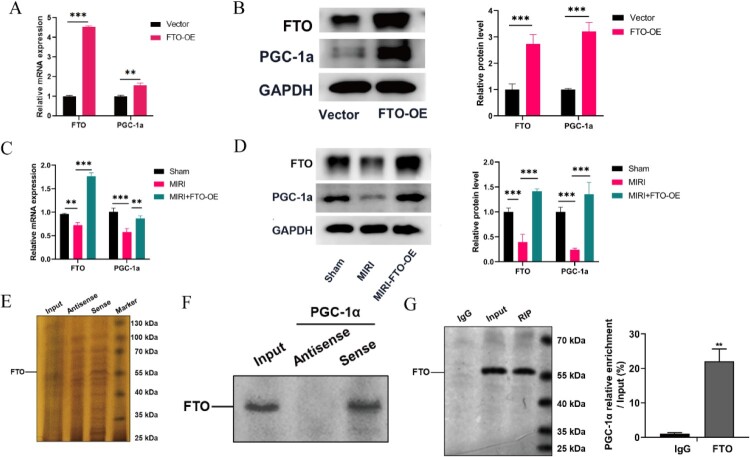
Identification of PGC-1a as a downstream target of FTO. (A) FTO and PGC-1a mRNA expression in FTO overexpression and normal H/R induced H9C2 cells (*N* = 3). (B) FTO and PGC-1a protein expression in FTO overexpression and H/R induced H9C2 cells (*N* = 3). (C) FTO and PGC-1a mRNA expression in sham, MIRI, and MIRI + FTO-OE rats (*N* = 3). (D) FTO and PGC-1a protein expression in sham, MIRI, and MIRI + FTO-OE rats (*N* = 3). (E) Silver staining of the gel after SDS‒PAGE separation of the proteins that were immunoprecipitated with the 3′ biotin-labeled probe for PGC-1a and the biotinylated antisense probe in RNA pull-down assays (*N* = 3). The line shows the position of FTO. (F)Western blot analysis following the RNA pull-down assay indicated the interaction between FTO and PGC-1a (*N* = 3). (G) RT‒PCR analysis following the RIP assay confirmed the interaction between FTO and PGC-1a (*N* = 3). **p < 0.01, ***p < 0.001. All the data were presented as the means ± SD.

Both RT–PCR and Western blot indicated that PGC-1a was accompanied by the expression of FTO in vitro (Figure 5**(**A, B)) and in vivo (Figure S1 A–C). Also, similar results were obtained in MIRI rats (Figure 5**(**C, D)). RNA pulldown assay and the immunoblot analysis confirmed the direct binding of FTO and PGC-1a (Figure 5**(**E, F)). RIP-PCR analysis also revealed that the PGC-1a mRNA was enriched by the anti-FTO antibody (Figure 5**(**G)).

### 3.6. FTO enhanced the stability of PGC-1a mRNA through uninstalling the m6A modification of PGC-1a mRNA

**Figure 6.**
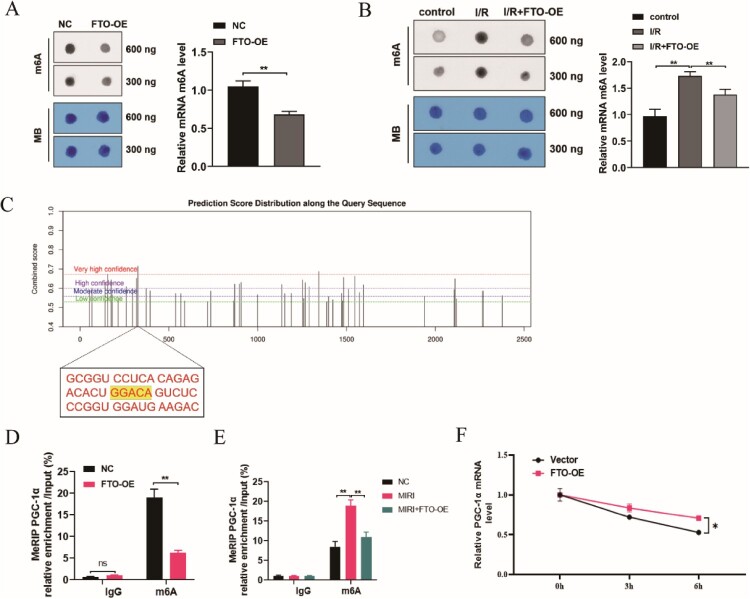
FTO enhanced the stability of PGC-1a mRNA through uninstalling the m6A modification of PGC-1a mRNA. (A) m6A dot blot analysis detected the m6A content in total RNA of H/R (Vector) and H/R + FTO-OE cardiomyocytes (*N* = 3). (B) m6A dot blot analysis detected the m6A content in total RNA of sham, MIRI, and MIRI + FTO-OE rats (*N* = 3). (C) Online predictive tool SRAMP showed the potential m6A modification sites and the m6A modification sites of PGC-1a mRNA were identified in the genomic sequence (GGACA). (D) MeRIP-PCR assay indicated the m6A modification level of H/R-induced cardiomyocytes upon FTO overexpression (*N* = 3). (E) MeRIP-PCR assay indicated the m6A modification level of sham, MIRI, and MIRI + FTO-OE rats (*N* = 3). (F) RNA stability analysis detected PGC-1a mRNA expression in H/R-induced cardiomyocytes treated with actinomycin D (*N* = 3). *p < 0.05, **p < 0.01. All the data were presented as the means ± SD.

Then, we analyzed the m6A modification level of PGC-1a in MIRI mice and H/R-induced cardiomyocytes. m6A dot blot analysis indicated that m6A modification levels were upregulated in MIRI rats (Figure 6**(**A)), but downregulated in FTO overexpression MIRI rats and H/R-induced cardiomyocytes (Figure 6A, B)). These results suggested a high m6A modification status in MIRI but was reversed by FTO overexpression. An online bioinformatics tool SRAMP (http://www.cuilab.cn/sramp) revealed that there are several potential m6A modification sites in the 3′-UTR with very high probability. The m6A modification sites of PGC-1a mRNA was GGACA (Figure 6**(**C)). MeRIP-qPCR assay indicated that PGC-1a m6A modification level was elevated in MIRI rats (Figure 6**(**D)). However, FTO overexpression reduced the PGC-1a m6A modification level in MIRI rats and H/R-induced cardiomyocytes (Figure 6**(**D,E)). The RNA stability analysis found that FTO overexpression increased the PGC-1a mRNA expression in H/R-induced cardiomyocytes treated with actinomycin D (Figure 6**(**F)). These results indicated that FTO enhanced the stability of PGC-1a mRNA through uninstalling the m6A modification of PGC-1a mRNA.

## 4. Discussion

In this study, we explored the role of FTO, a key regulator of m6A RNA methylation, in MIRI and H/R injury in cardiomyocytes. The findings suggested that FTO played a protective role in MIRI and H/R-induced myocardial injury by modulating oxidative stress, mitochondrial function, and the stability of PGC-1α mRNA. Specifically, FTO overexpression mitigated myocardial damage, reduced ROS accumulation, and promoted mitochondrial biogenesis, highlighting its potential as a therapeutic target for ischemic heart disease.

Previous studies have established that oxidative stress and mitochondrial dysfunction are key contributors to the pathophysiology of ischemic heart disease and other cardiovascular disorders [24]. In our experiments, we observed that FTO overexpression mitigated excessive oxidative stress and enhanced mitochondrial biogenesis in both H/R-treated cardiomyocytes and MIRI rats, indicating that FTO may regulate mitochondrial health in response to ischemic stress. This finding is consistent with recent reports that highlights the importance of FTO in maintaining cellular homeostasis, including its involvement in the regulation of mitochondrial dynamics [25]. In hepatic ischemia–reperfusion injury (HIRI), FTO expression was reduced, and its overexpression alleviated HIRI by reducing m6A methylated RNA levels, mitigating oxidative stress, and preventing mitochondrial fragmentation both in vivo and in vitro [26]. Furthermore, FTO exerted its protective effect by demethylating Drp1 mRNA and inhibiting Drp1-mediated mitochondrial fragmentation [26]. FTO has been shown to augment the proliferation and metastasis of gastric cancer by modulating mitochondrial fission/fusion dynamics and metabolic processes, which involves the facilitation of caveolin-1 mRNA degradation via demethylation [27]. FTO reduced mitochondrial ROS, restored respiratory function, and maintained mitochondrial membrane potential by modulating BNIP3 transcript expression via an m6A-dependent mechanism [28]. Silencing FTO increased m6A modification in BNIP3 coding regions, promoting YTHDF2 binding, mRNA destabilization, and a decrease in BNIP3 protein levels [28].

Our identification of PGC-1α as a downstream target of FTO underscored the critical role of this protein in regulating mitochondrial biogenesis. PGC-1α is a master regulator of mitochondrial function, and its stability is crucial for the maintenance of mitochondrial integrity [29]. In mice with acute kidney injury (AKI), the expression of the protein PGC-1α was diminished and overexpression of PGC-1α subsequently activated TFEB-mediated autophagy, which led to the amelioration of mitochondrial dysfunction and kidney damage [30]. Intrauterine prenatal hypoxia–ischemia (HI) activated the PGC-1α-NRF-1-TFAM signaling pathway, resulting in mitochondrial dysfunction [31]. Subsequent postnatal administration of pioglitazone enhanced PGC-1α expression and mitochondrial biogenesis, thereby mitigating hippocampal injury and associated cognitive deficits [31]. In this study, we found that FTO enhanced the stability of PGC-1α mRNA by removing m6A modifications, a process that may contribute to the restoration of mitochondrial biogenesis and mitigation excessive oxidative stress in H/R-treated cardiomyocytes and MIRI rats. Furthermore, it is important to consider whether all the cellular responses observed in this study can be attributed solely to PGC-1α. While PGC-1α is a key regulator of mitochondrial function, it is unlikely to be the only mediator of the effects of FTO in our model. Other downstream targets and regulatory pathways may also contribute to the observed cellular protection. FTO influences specific cardiac pathways, including those associated with sarcomere organization, myofibril assembly, calcium handling, and contractility [17,32]. Furthermore, it also affects pathways involved in angiogenesis and extracellular matrix (ECM) fibrosis, as well as long non-coding RNAs (lncRNAs) that are implicated in fibrosis and hypertrophy [17,32]. HIF1α, activated by myocardial infarction (MI)/hypoxia, bound to the FTO promoter to decrease its expression, causing aberrant m6A modification, while FTO-mediated m6A modification of EPRS, regulated by IGF2BP3, played a key role in cardiac fibrosis after MI [33]. Additional mechanisms involving FTO-regulated pathways and its interactions with other transcription factors, may also contribute to the observed protective effects. Further studies will be needed to elucidate the full spectrum of FTO’s regulatory roles in cardiac ischemic injury and its potential as a therapeutic target for ischemic heart diseases.

## 5. Conclusions

In summary, our research uncovered the role of m6A demethylase FTO in modulating oxidative stress and mitochondrial biogenesis in H/R-induced cardiomyocytes and MIRI rats. Specifically, FTO was shown to decrease the m6A modification of PGC-1α mRNA, leading to increased stability and expression of this key regulator. These discoveries offer a promising new perspective for developing therapeutic approaches to address MIRI.

## Author contributions

Qiong Jiang, Lianglong Chen and Feilong Zhang designed the study. Qiong Jiang, Xuehai Chen, Kezeng Gong and Zhe Xu contributed to the animal experiments, cell experiments, and data analysis. Qiong Jiang wrote the final manuscript. Xuehai Chen, Lianglong Chen and Feilong Zhang revised the manuscript. All authors read and approved the final manuscript.

## Supplementary Material

Supplemental Material.docx

## Data Availability

Data will be made available on request.
