# Supplementary material for: M6a demethylase FTO regulates the oxidative stress, mitochondrial biogenesis of cardiomyocytes and PGC-1a stability in myocardial ischemia-reperfusion injury
Source: Redox Rep. 2025 Jan 27;30(1):2454892. doi: 10.1080/13510002.2025.2454892 (PMC11774161; doi:10.1080/13510002.2025.2454892)
Supplement: Supplemental Material.docx [file YRER_A_2454892_SM3848.docx]

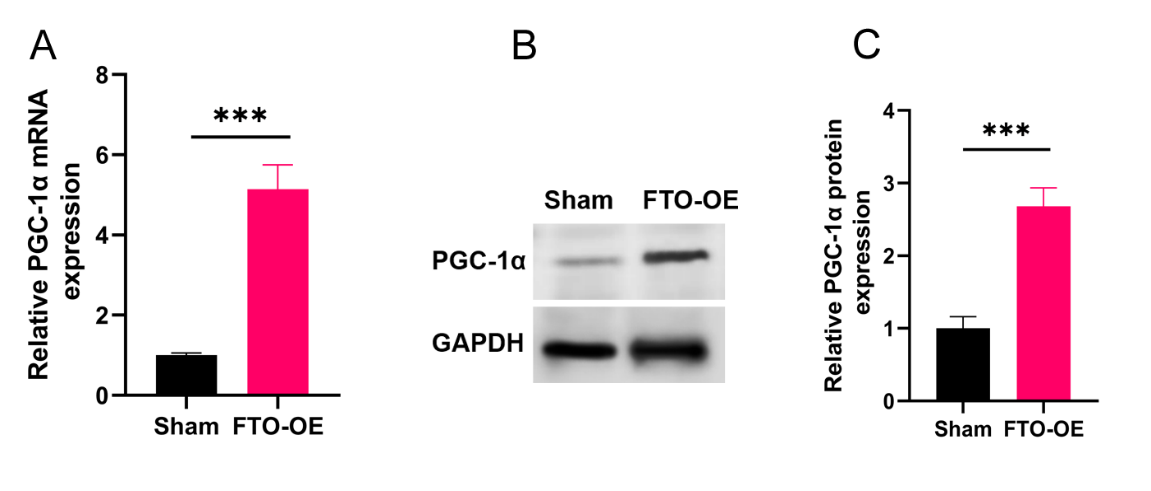


Figure S1. RT-PCR(A) and Western blot (B-C) assay were performed to determine the expression of PGC-1α in FTO-OE rats (N=3). ***p<0.001. All the data were presented as the means ± SD.
